# Measuring office workplace interactions and hand hygiene behaviors through electronic sensors: A feasibility study

**DOI:** 10.1371/journal.pone.0243358

**Published:** 2021-01-19

**Authors:** Paul N. Zivich, Will Huang, Ali Walsh, Prabal Dutta, Marisa Eisenberg, Allison E. Aiello

**Affiliations:** 1 Department of Epidemiology, University of North Carolina at Chapel Hill, Chapel Hill, North Carolina, United States of America; 2 Carolina Population Center, Chapel Hill, North Carolina, United States of America; 3 College of Engineering, University of California, Berkley, California, United States of America; 4 Department of Systems, Populations, and Leadership, University of Michigan School of Nursing, Ann Arbor, Michigan, United States of America; 5 Department of Epidemiology, University of Michigan, Ann Arbor, Michigan, United States of America; The University of Hong Kong, CHINA

## Abstract

Office-based workplaces are an important but understudied context for infectious disease transmission. We examined the feasibility of two different sensors (Opos and Bluetooth beacons) for collecting person-to-person contacts and hand hygiene in office-based workplaces. Opo is an interaction sensor that captures sensor-to-sensor interactions through ultrasonic frequencies, which correspond to face-to-face contacts between study participants. Opos were additionally used to measure hand hygiene events by affixing sensors to soap and alcohol-based hand sanitizer dispensers. Bluetooth beacons were used in conjunction with a smartphone application and recorded proximity contacts between study participants. Participants in two office sites were followed for one-week in their workplace in March 2018. Contact patterns varied by time of day and day of the week. Face-to-face contacts were of shorter mean duration than proximity contacts. Supervisors had fewer proximity contacts but more face-to-face contacts than non-supervisors. Self-reported hand hygiene was substantively higher than sensor-collected hand hygiene events and duration of hand washing events was short (median: 9 seconds, range: 2.5–33 seconds). Given that office settings are key environments in which working age populations spend a large proportion of their time and interactions, a better characterization of empirical social networks and hand hygiene behaviors for workplace interactions are needed to mitigate outbreaks and prepare for pandemics. Our study demonstrates that implementing sensor technologies for tracking interactions and behaviors in offices is feasible and can provide new insights into real-world social networks and hygiene practices. We identified key social interactions, variability in hand hygiene, and differences in interactions by workplace roles. High-resolution network data will be essential for identifying the most effective ways to mitigate infectious disease transmission and develop pandemic preparedness plans for the workplace setting.

## Introduction

Employed individuals in the United States worked an average of 8.3 hours per day during the week, and the majority (82%) of employed individuals did some or all of their work in an office or place of employment in 2018 [1]. Prior research suggests almost a fifth of known contacts with visibly ill individuals occurred in the workplace and these workplace contacts have been associated with an increased risk of gastrointestinal and respiratory illness [2]. Thus, the office environment is an important, yet understudied, setting for infectious disease transmission and prevention. Despite the relevance of interactions in office-based workplaces in infectious disease transmission and epidemic patterns; empirical data and research on office interactions, contact patterns, and health behaviors are lacking. Our recent systematic review on hand hygiene in office-based workplaces found no studies that measured human contact and interactions in these settings [3]. This knowledge is particularly relevant in the context of the SARS-CoV-2 pandemic. Given the potential risk, the SARS-CoV-2 pandemic has shuttered numerous workplaces worldwide [4]. Furthermore, scant data and knowledge about contact patterns and pathogen transmission have made public perception of re-opening non-essential workplaces fraught with uncertainty [5].

Contact tracing, or the mapping of close in-person contacts, has been previously conducted via self-reported surveys and sensor-based technologies. However, interaction data collected via self-reported surveys are prone to underreporting, particularly for contacts of short duration [6]. Recent studies have suggested that using sensors to measure contact networks can improve contact data quality by capturing contacts with high granularity [7–10]. Nonetheless, there are still significant barriers related to measurement of interaction through the use of electronic sensors. Electronic sensors vary by costs, technological restrictions (e.g., interactions must fall within a strict duration and distance definition to be captured by a given sensor, etc.), and data quantity versus quality [11].

Infectious disease dynamics are dependent on a number of factors including contact patterns, and individual attributes and behaviors (e.g., hand hygiene, etc.) [12, 13]. In particular, social mixing can modify the impact of individual factors on population dynamics [14]. Therefore, accurate characterizations of individual health behaviors and contact patterns for office workplaces is important. Observational studies have found that self-reported hand hygiene is prone to measurement error, with people overestimating the number of times they wash their hands or use hand sanitizer and the duration of handwashing [15]. Therefore, measurement of hand hygiene events through sensors has potential to reduce bias [16]. Furthermore, active monitoring of hand hygiene (with or without reminders for hand hygiene moments) has been shown to increase optimal hand hygiene in hospital settings [17, 18], suggesting that hand hygiene monitoring can also be used as an intervention to boost compliance.

With the knowledge that contacts in offices play a role in infectious disease transmission, structures of the contact network influence how infectious are transmitted, and self-reported contacts may have substantial measurement error; assessments of alternative methods for network collection are needed. Larger data collection can be undermined if participants do not find the methods of data collection to be acceptable, participants are not adequately incentivized, or sensors are difficult to implement. While previous work has found electronic sensors to collect contacts as acceptable; those studies were conducted in substantively different settings (i.e.; epidemiology conference [8], high school [7, 10], middle school [9]), implemented for short durations (followed only for one day [7–9]), and used different technologies. Therefore, the practicality of collecting contacts in office settings with electronic sensors is unknown.

We conducted a longitudinal pilot study to assess the feasibility of using contact sensors for collecting empirical social network data in common US office settings. The overall aims of this study were to: 1) assess two sensor technologies for collecting contact data in office settings; 2) describe and characterize workplace in-person contact patterns; and 3) measure hand hygiene in office environments. This manuscript describes the study design and implementation of the sensor technologies, as well as the sensor-collected contact and hand hygiene data and participant feedback from two prototypical offices sites in the US.

## Methods

We conducted a pilot longitudinal network study of contact patterns and hand hygiene in two Midwestern offices (designated as site A and site B hereafter) in the United States during March 2018. The office worksites were chosen based on convenience but comprise typical layouts of many office workplaces in the US; including open-air cubicles, private offices, bathrooms, and shared breakroom space. Participants were recruited via email through the company listserv, posters in common areas, and in-person by study staff. To be eligible, employees needed to be at least 18 years old and plan to work in the office for at least part of the follow-up period. At each office site, individuals were enrolled and followed during working hours for one work-week (Monday 10am to Friday 3pm) during consecutive weeks during March 2018. At enrollment, participants provided informed consent, completed an online baseline survey, and issued two sensors for the study period. At the end of the study period, participants responded to an exit survey and were given a $25 gift card as an incentive for participation. This data collection and research were approved by the University of North Carolina Institutional Review Board and all participants provided written consent.

### Surveys

At baseline, participants reported the following demographic and employment information: age (years), gender (male; female), race (white; black; Asian; Native Hawaiian or Other Pacific Islander; American Indian or Alaskan Native; multi-racial; other), ethnicity (Hispanic or Latino; non-Hispanic), marital status (currently married; never married; separated; divorced; widowed; living with a partner), education level (high school graduate; some college; college graduate; post-graduate), type of office they worked in (individual office; shared office; cubicle; desk in an open location), and whether their occupational role included supervising others (yes; no). In the exit survey, participants were asked regarding the usage and the acceptability of the two sensors. In both baseline and exit surveys, participants were asked to report how many times they washed their hands during the previous work day. Participants were also asked about their use of hand sanitizer.

### Contact sensors

#### Person-to-person interactions

Opo is an interaction distance sensor that measures distance (within 2 meters) to other Opos through ultrasonic (40,000 Hz) frequencies at a time resolution of 5 seconds [19]. Opos capture interactions or contacts with other Opos when the front of sensors are facing each other, rather than capturing general proximity contracts. When Opos are worn on the front of an individual, sensor interactions represent face-to-face contacts within a distance of two meters. This allows the researcher to identify face-to-face contacts between individuals wearing Opos and interactions with objects outfitted with Opos. Data is stored on the sensor and is downloaded to a computer. Participants were asked to wear the Opo sensor during their entire time at the office for the one-week period. Opos were collected several times over the study period to have their batteries charged and data downloaded to assess data quality. At site B, several floors of the building were equipped with ultrasonic motion detectors connected to the indoor lighting, which used the same ultrasonic frequencies as the Opos. As such, contacts were only collected for a participants on a single floor where no interference from the ultrasonic motion sensors occurred (n = 8, 40%).

Proximity contact data was collected using a second contact sensor using Bluetooth Low Energy (BLE) beacon technology. Participants were given a BLE beacon to carry during their workday and asked to download the Ethica (Ethica Data, https://ethicadata.com/) smartphone application on their personal smartphone. While the Ethica application is running, it collects nearby BLE broadcasts that correspond to a unique study identifier. Due to smartphone manufacturer constraints, the time resolution of the BLE contact data is five minutes. Unlike the Opo which collects face-to-face contacts, BLE contact data reflects general proximity contacts. For BLE interactions to be recorded, at least one individual must be carrying their smartphone running the Ethica application and the other must be carrying their beacon. The BLE collects received signal strength indicator (RSSI) data, which is a unitless measure of signal strength that provides some indication of distance and presence of physical barriers between sensors. Since the BLE requires participants to have a smartphone to download the Ethica application, only participants that had a smartphone capable of installing the application were included in the BLE component of the study (site A: n = 18, 78%, site B: n = 20, 100%). The beacons used as part of this study had a battery life over one month. All information was downloaded from the Ethica servers at the end of the follow-up.

#### Hand hygiene measurement

In order to measure workplace hand hygiene, soap and alcohol sanitizer dispensers in the offices were also equipped with Opos. We placed 19 Opos in restrooms that were on the same floor as the participant offices/cubicles as well as in public areas at site A. Additionally, 3 Opos were affixed to alcohol sanitizer dispensers in common areas. Opos affixed to soap and alcohol sanitizer dispensers were fitted with an accelerometer to detect vibrations, indicative of dispenser use. All bathrooms at site B were equipped with ultrasonic motion sensors that used the same ultrasonic frequencies as the Opos. As a result, no usable hand hygiene data was collected from site B.

### Descriptive analyses

We described distributions of demographic characteristics by study site through counts and percentages; or median, 25^th^, and 75^th^ percentiles. Variables representing race, ethnicity, education, and marital status categories were collapsed, given small cells sizes for some categories. Self-reported hand hygiene data was expressed through median and percentiles. Sensor-collected hand hygiene was summarized through counts of unique events per participant. Additionally, time spent performing hand hygiene in seconds was calculated from the interaction times between the participant’s sensor and dispenser sensor. Total time spent for the hand hygiene event was calculated from the initial contact to 2.5 seconds after the final contact, with a 7.5 second “grace period” between recorded interactions to account for the time-resolution of the Opo sensor.

Person-to-person contact data from the Opo and BLE sensors was used to generate contact networks between study participants that occurred during the workday. We visualized weekly, daily, and hourly networks by sensor and office site as heatmaps of the adjacency matrices for contacts. The colors of the squares were determined by the duration of contact between the two participants. Unique contacts were defined as the existence of any between two participants over follow-up. Density was defined as the actual unique contacts divided by the maximum possible unique contacts over the entire follow-up. Opo contact durations were calculated via the difference between an initial contact and 2.5 seconds after the final contact, with a 7.5 second grace period between recorded interactions between participant sensors. For BLE contact durations, contact durations were similarly calculated as the difference between the initial and final contact, but with an additional 2.5 minutes after the final contact and a 5.5 minute grace period between interactions. All analyses and visualizations were generated using Python v3.5.2 (Python Software Foundation, https://www.python.org/).

## Results

### Sample characteristics

We recruited a convenience sample of 23 individuals from site A and 20 individuals from site B. A majority of participants were white (A: 87%, B: 85%) and currently married (A: 65%, B: 70%). Employees at site A were older, had higher levels of education, and worked more in individual offices compared to site B (Table 1).

**Table 1:** Self-reported characteristics of participants at office sites A and B.

		Site A (n = 23)		Site B (n = 20)	
		n / median	% / IQR	n / median	% / IQR
Age		46	39, 55	34	28, 43
Female		10	43%	7	35%
Race / ethnicity					
	White, non-Hispanic	20	87%	17	85%
	Other	3	13%	3	15%
Currently married		15	65%	14	70%
Education level					
	Some college or less	3	13%	3	15%
	College graduate or more	20	87%	17	65%
Office type					
	Individual office	12	52%	6	30%
	Shared office	2	9%	2	10%
	Cubicle	9	39%	12	60%
Supervisor		7	30%	9	45%

IQR: interquartile range

Offices were located in the Midwestern United States. Offices were chosen based on convenience but share features of typical US workplaces; including open-air cubicles, private offices, bathrooms, and shared breakroom space. At each site, individuals were enrolled and followed for one work-week (Monday 10am–Friday 3pm) during working hours during consecutive weeks in March 2018.

### Recorded contacts

**Fig 1:**
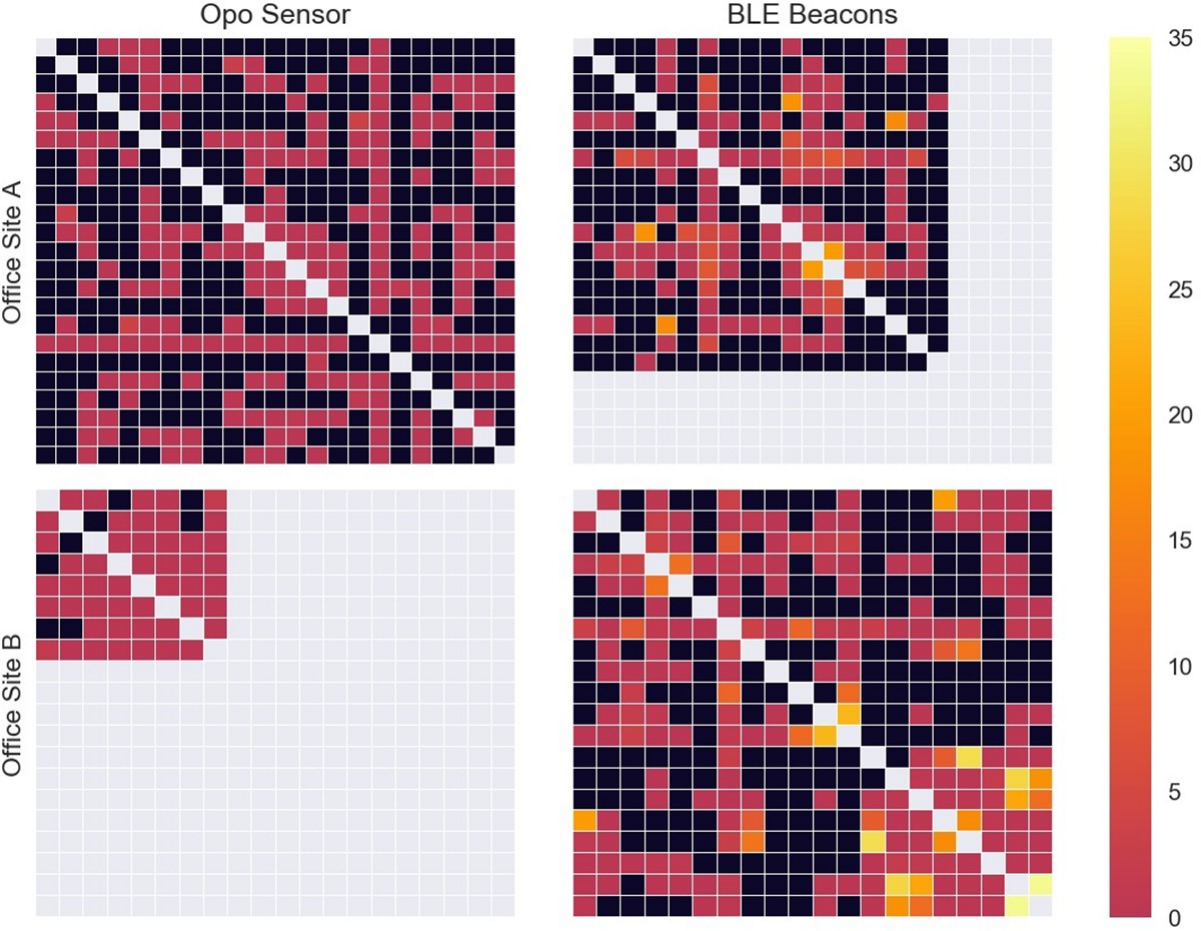
Weekly contact data for office sites A and B by sensor. Light gray squares indicate participants who did not use the indicated sensors. Black squares indicate no collected contacts between participants. The heatmap scale indicates duration of contacts in hours.

**Fig 2:**
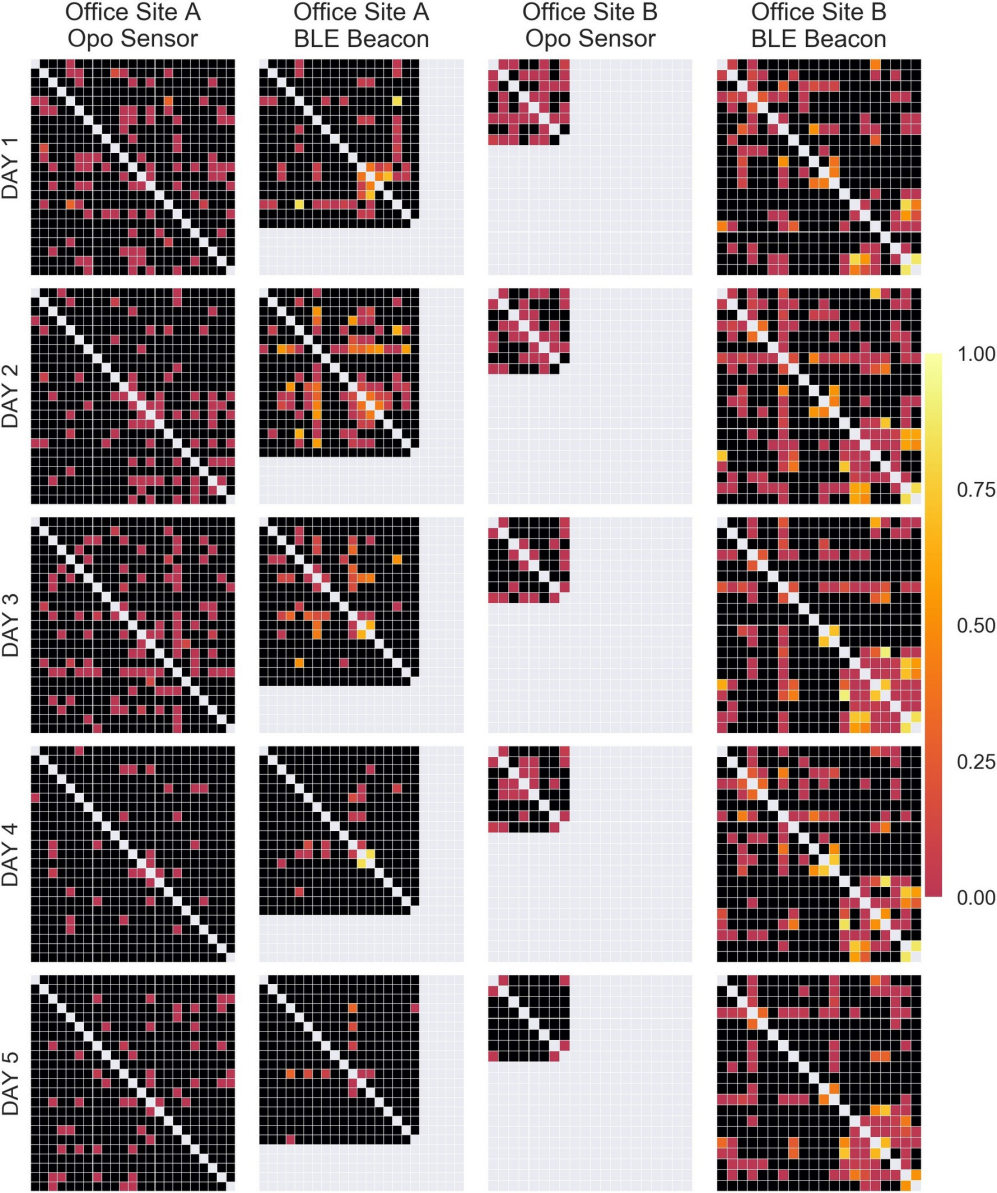
Daily contact data for office sites A and B by sensor. Light gray squares indicate participants who did not use the indicated sensors. Black squares indicate no collected contacts between participants. The heatmap scale indicates the proportion of time spent in contact relative to the total number of hours of follow-up for that day.

For site A, 23 individuals were provided Opos and 18 (78%) were given BLE beacons. Three individuals did not work from the office on Thursday and Friday. For site B, all 20 individuals were given both Opos and BLE beacons, and all employees worked from the office for the study period. Due to interference from the ultrasonic motion detectors, Opo contact data was only available for 8 participants at site B. Overall interactions for both sites are displayed in Fig 1, and daily interactions are displayed in Fig 2. Dynamic visualizations of contacts by hour are available in the online S1 and S2 Videos supplements. As seen in across visualizations, BLE sensors recorded longer durations of contacts.

At site A, there were 103 unique contacts (density: 0.41) with the Opo and 66 (density: 0.43) with the BLE sensors. The median recorded total time in contact between individuals for the entire study period was 0.1 minutes (IQR: 0.0–0.3, Range: 0.0–130.7) for the Opo and 29.6 minutes (IQR: 7.5–171.1, Range: 2.5–502.0) for BLE. Supervisors at site A had a more contacts recorded by Opo (mean: 9.6) compared to non-supervisors (mean: 8.7). For BLE sensors, the reverse was observed for supervisors (mean: 4.2) and non-supervisors (mean: 8.5).

At site B, there were 24 unique contacts (density: 0.86) with the Opo and 111 (density: 0.58) with the BLE sensors. The median recorded total time in contact for the study period was 0.5 minutes (IQR: 0.1–2.0, Range: 0.0–34.2) for the Opo and 12.9 minutes (IQR: 5.0–181.8, Range: 2.5–538.5) for the BLE. Similar to site A, supervisors had more unique contacts compared to non-supervisors for both Opo (mean for supervisor: 6.3, mean for non-supervisor: 5.8) and BLE sensors (mean for supervisor: 9.7, mean for non-supervisor: 12.3).

### Hand hygiene

Self-reported hand hygiene is shown in Table 2. At site A, there were 14 participants who self-reported hand hygiene with at least one sensor-measured event at the soap dispenser. Individuals self-reported washing their hands 5 times (median: 5, IQR: 4–6), but the median hand hygiene frequency captured by Opos affixed to soap dispensers was once (IQR: 1–2). Nine (39%) participants had no sensor-recorded hand hygiene events over the study duration. Over the week, 3 (13%) had three recorded events, 4 (17%) had two recorded events, and 7 (30%) had one recorded event. Of recorded soap dispenser interactions, they were often of short recorded duration (median: 9 seconds, IQR: 6–14, Range: 2.5–33). No sensor-collected hand hygiene events were available at site B due to interference from the ultrasonic motion detectors. For alcohol-based dispensers, only seven interactions were captured, of which two individuals had three interactions and one had one interaction.

**Table 2:** Self-reported hand hygiene of participants at two office sites.

			Site A (n = 23)			Site B (n = 20)		
			median	IQR	range	median	IQR	range
Self-reported daily hand washing with soap								
	Baseline		5	4, 6	2, 12	4.5	3, 5.25	1, 8
	End of follow-up		4	3, 5	2, 10	3.5	3, 5	1, 6
		Missing*	10			4		
Self-reported daily alcohol-based sanitizer use								
	Baseline		0	0, 1	0, 3	0	0, 2	0, 4
	End of follow-up		0	0, 0	0, 2	1	0, 2	0, 5
		Missing*	10			4		

Participants were asked to reported how many times they washed their hands (or used hand sanitizer) during the previous work day.

* Data missing for the end of follow-up

### Sensor feedback

At the end of follow-up, participants were asked about the usage of the sensor over the week (n = 28). Most participants reported wearing their assigned Opo sensor the entire study period (79%) or most of the time (18%), with one-person reporting wearing the sensor for only 4 days. For the BLE sensor, most participants reported carrying the BLE beacon with them for the entire study period (57%) or most of the study period (32%). App use followed a similar pattern (entire study period: 50%, most of the study period: 36%). The self-reports of sensor use are generally supported by the interaction data collected over the study period.

We further asked participants about usage of the sensors through a comment section on the survey. In general, most participants considered Opo sensors easy to use. Some participants had issues with the size of the Opos, found a light on the sensor distracting, or felt that the location where the sensor had to be worn was bothersome. Participants also found the BLE beacons relatively easy to use. Some issues with BLE beacons included draining phone battery, forgetting to start the application when returning to work, and a few participants had issues related to running the application on their phones. Regarding both sensors, participants stated they would be willing to participate over a longer study duration. However, some participants stated they would need an increase in compensation for a longer study period.

## Discussion

To our knowledge, this is the first study to collect workplace interactions and hand hygiene instances within the workplace. Contacts were collected through sensors technologies that used ultrasonic frequencies and Bluetooth. Participants felt that using the sensors was simple and were willing to have their interactions tracked in an office setting. Additionally, we were able to identify some limitations of the sensors related to appearance and functionality. We also found that there are some structural technologies in the workplace, such as ultrasonic motion detectors, that can interfere with some sensors, suggesting multiple sensors that use different technologies should be used when possible. Furthermore, sensors should be selected based on the contact types that are deemed most important in regards to the study question of interest.

While the two offices differed in composition of office types and overall layout, the network structures between worksites shared some similarities. The networks visualizations produced from this study, suggest that face-to-face interactions commonly occur in the workplace and that the frequency of interaction varied by job role. If the structure of the contact network in office settings does vary by job roles, this may have implications for interventions on improving hand hygiene. For example, target interventions by job roles may more effectively interrupt transmission or be more cost-effective to implement. Sensor-collected proximity contacts were of substantially longer duration than sensor-collected face-to-face contacts, suggesting that many contacts were comprised of individuals being in the same vicinity but not actually facing each other.

We also noted that individuals self-reported higher levels of hand hygiene on surveys versus the measures collected by the Opo sensor. These findings are consistent with other electronic sensors in the hospital setting for measuring hand hygiene events [20, 21]. In hospitals, staff often overestimate the number of times that they wash or sanitize their hands [15, 20, 22, 23]. Similar patterns of overreporting hand hygiene have been observed among university students as well [24, 25]. Reasons for this overestimation are unclear, but could be related to reporting biases, such as social desirability bias [26], where individuals feel that they need to report that they are washing their hands more because it is socially desirable to be hygienic [27]. Alternatively, the reporting may be accurate because it could have included both hand hygiene events that occurred in the office as well as outside of the office. Relatedly, individuals that washed their hands in venues not fitted with the Opo would remain undetected. While we outfitted bathrooms most likely to be used, participants could have washed their hands in off-site restrooms. Alternatively, if Opo sensors were not worn or worn improperly (i.e. not facing the soap dispenser affixed with Opos) the hand hygiene event would not be recorded.

While we were able to identify and visualize participant interactions in the offices, there is the potential for measurement error of contacts for each of the sensors in this study. First, the Opo sensor may miss contacts if the sensors were not aligned during face-to-face interaction or if they were obstructed (e.g., by a jacket or lanyard). While the BLE beacon was able to capture both face-to-face and proximity interactions, it is not possible to distinguish between these types of interactions. Furthermore, the BLE beacon does not provide a reliable measure of interaction distance. Additionally, false positive contacts are possible due to the signal strength of BLE beacons (i.e. Bluetooth signals travelling through walls and identifying a participant in another office as a contact). While not used in our analysis, filtering detected contacts by RSSI values may limit these false positives. In addition, the BLE beacon time resolution (5 minutes) makes it difficult to identify short contacts due to their transient nature. The concern of false negatives is lessened for Opo sensors, since their signals do not travel through walls. While we collected networks primarily through electronic sensors, there are alternative approaches to collecting network data including; line-lists, records of meetings, geographical proximity, and direct observations. Ultimately, the research question should guide the method used to collect contact data in the workplace. In the future, we recommend the use of at least two approaches, where the chosen methods compensate for errors in the other.

## Conclusion

We conducted a feasibility study for collecting personal interactions and hand hygiene instances in the office environment using two different sensor technologies. The sensors identified numerous interactions between individuals and use of hand hygiene dispensers. We found that reported hand hygiene events may be overestimated and that interactions through both face-to-face and within the general vicinity, are common in the workplace setting. We also found that those with higher level supervisory roles had fewer long proximity interactions compared to those without supervisor responsibilities, suggesting that roles in the workplace may lead to differing patterns of infectious disease transmission in the workplace. Given the role of the work environment as an integral aspect of pandemic planning, future work should focus on implementing sensor technologies in larger office populations to map out and link workplace interactions, hand hygiene instances, and transmission of infectious diseases. Together, these types of data will be crucial for identifying the most effective ways to mitigate transmission of infectious diseases and pandemic threats in workplace populations.

## Supporting information

S1 File Details on implemented sensors. (XLSX)

S1 Video Hourly contact data for office site A by sensor. Light gray squares indicate participants who did not use the indicated sensors. Black squares indicate no collected contacts between participants. The heatmap scale indicates the proportion of time spent in for that hour.
(MP4)

S2 Video Hourly contact data for office site B by sensor. Light gray squares indicate participants who did not use the indicated sensors. Black squares indicate no collected contacts between participants. The heatmap scale indicates the proportion of time spent in for that hour.
(MP4)

S1 Data (DOCX)

## References

[1] American Time Use Survey Summary [Internet]. Bureau of Labor Statistics; 2019; June 19, 2019 [cited September 22, 2019]. Available from: https://www.bls.gov/news.release/atus.nr0.htm

[2] Hovi T, Ollgren J, Haapakoski J, Amiryousefi A, Savolainen-Kopra C. Exposure to persons with symptoms of respiratory or gastrointestinal infection and relative risk of disease: self-reported observations by controls in a randomized intervention trial. Trials. 2015;16:168 Epub 2015/04/17. 10.1186/s13063-015-0691-4 25879224 PMC4412200

[3] Zivich PN, Gancz AS, Aiello AE. Effect of hand hygiene on infectious diseases in the office workplace: A systematic review. American journal of infection control. 2018;46(4):448–55. Epub 2017/12/03. 10.1016/j.ajic.2017.10.006 . 29195781

[4] Gostin LO, Wiley LF. Governmental Public Health Powers During the COVID-19 Pandemic: Stay-at-home Orders, Business Closures, and Travel Restrictions. Jama. 2020;323(21):2137–8. 10.1001/jama.2020.5460 32239184

[5] Czeisler MÉ, Tynan MA, Howard ME, Honeycutt S, Fulmer EB, Kidder DP, et al Public attitudes, behaviors, and beliefs related to COVID-19, stay-at-home orders, nonessential business closures, and public health guidance—United States, New York City, and Los Angeles, May 5–12, 2020. Morbidity and Mortality Weekly Report. 2020;69(24):751 10.15585/mmwr.mm6924e1 32555138 PMC7302477

[6] Smieszek T, Burri EU, Scherzinger R, Scholz RW. Collecting close-contact social mixing data with contact diaries: reporting errors and biases. Epidemiology and infection. 2012;140(4):744–52. Epub 2011/07/08. 10.1017/S0950268811001130 . 21733249

[7] Smieszek T, Barclay VC, Seeni I, Rainey JJ, Gao H, Uzicanin A, et al How should social mixing be measured: comparing web-based survey and sensor-based methods. BMC infectious diseases. 2014;14:136 Epub 2014/03/13. 10.1186/1471-2334-14-136 24612900 PMC3984737

[8] Smieszek T, Castell S, Barrat A, Cattuto C, White PJ, Krause G. Contact diaries versus wearable proximity sensors in measuring contact patterns at a conference: method comparison and participants' attitudes. BMC infectious diseases. 2016;16:341 Epub 2016/07/28. 10.1186/s12879-016-1676-y 27449511 PMC4957345

[9] Leecaster M, Toth DJ, Pettey WB, Rainey JJ, Gao H, Uzicanin A, et al Estimates of Social Contact in a Middle School Based on Self-Report and Wireless Sensor Data. PloS one. 2016;11(4):e0153690 Epub 2016/04/23. 10.1371/journal.pone.0153690 27100090 PMC4839567

[10] Mastrandrea R, Fournet J, Barrat A. Contact Patterns in a High School: A Comparison between Data Collected Using Wearable Sensors, Contact Diaries and Friendship Surveys. PloS one. 2015;10(9):e0136497 Epub 2015/09/02. 10.1371/journal.pone.0136497 26325289 PMC4556655

[11] Funk S, Bansal S, Bauch CT, Eames KTD, Edmunds WJ, Galvani AP, et al Nine challenges in incorporating the dynamics of behaviour in infectious diseases models. Epidemics. 2015;10:21–5. 10.1016/j.epidem.2014.09.005 25843377

[12] Ferguson N. Capturing human behaviour. Nature. 2007;446(7137):733 10.1038/446733a 17429381

[13] VanderWeele TJ, Christakis NA. Network multipliers and public health. International journal of epidemiology. 2019;48(4):1032–7. 10.1093/ije/dyz010 30793743 PMC6693811

[14] Read J, Edmunds W, Riley S, Lessler J, Cummings D. Close encounters of the infectious kind: methods to measure social mixing behaviour. Epidemiology & infection. 2012;140(12):2117–30. 10.1017/S0950268812000842 22687447 PMC4288744

[15] Jenner EA, Fletcher BC, Watson P, Jones F, Miller L, Scott G. Discrepancy between self-reported and observed hand hygiene behaviour in healthcare professionals. Journal of hospital infection. 2006;63(4):418–22. 10.1016/j.jhin.2006.03.012 16772101

[16] Mastrandrea R, Soto-Aladro A, Brouqui P, Barrat A. Enhancing the evaluation of pathogen transmission risk in a hospital by merging hand-hygiene compliance and contact data: a proof-of-concept study. BMC research notes. 2015;8(1):426. 26358118 10.1186/s13104-015-1409-0 PMC4566487

[17] Swoboda SM, Earsing K, Strauss K, Lane S, Lipsett PA. Electronic monitoring and voice prompts improve hand hygiene and decrease nosocomial infections in an intermediate care unit. Critical care medicine. 2004;32(2):358–63. Epub 2004/02/06. 10.1097/01.CCM.0000108866.48795.0F . 14758148

[18] Venkatesh AK, Lankford MG, Rooney DM, Blachford T, Watts CM, Noskin GA. Use of electronic alerts to enhance hand hygiene compliance and decrease transmission of vancomycin-resistant Enterococcus in a hematology unit. American journal of infection control. 2008;36(3):199–205. Epub 2008/03/29. 10.1016/j.ajic.2007.11.005 . 18371516

[19] Huang W, Kuo Y-S, Pannuto P, Dutta P. Opo: A Wearable Sensor for Capturing High-Fidelity Face-to-Face Interactions. In: Machinery AfC, editor. 12th ACM Conference on Embedded Networked Sensor Systems (SenSys '14); 11 3–6, 2014; Memphis, TN2014.

[20] Broughall JM, Marshman C, Jackson B, Bird P. An automatic monitoring system for measuring handwashing frequency in hospital wards. Journal of Hospital Infection. 1984;5(4):447–53. 10.1016/0195-6701(84)90016-1 6085102

[21] Ellingson K, Haas JP, Aiello AE, Kusek L, Maragakis LL, Olmsted RN, et al Strategies to prevent healthcare-associated infections through hand hygiene. Infection control and hospital epidemiology. 2014;35 Suppl 2:S155–78. Epub 2014/11/08. 10.1017/s0899823x00193900 . 25376074

[22] Larson EL, Aiello AE, Cimiotti J. Assessing nurses' hand hygiene practices by direct observation or self-report. 2004 15916321

[23] Seyed Nematian SS, Palenik CJ, Mirmasoudi SK, Hatam N, Askarian M. Comparing knowledge and self-reported hand hygiene practices with direct observation among Iranian hospital nurses. American journal of infection control. 2017;45(6):e65–e7. 10.1016/j.ajic.2017.03.007 28427787

[24] Surgeoner BV, Chapman BJ, Powell DA. University students’ hand hygiene practice during a gastrointestinal outbreak in residence: what they say they do and what they actually do. Journal of environmental health. 2009;72(2):24–9. 19761005

[25] Thumma J, Aiello AE, Foxman B. The association between handwashing practices and illness symptoms among college students living in a university dormitory. American journal of infection control. 2009;37(1):70–2. Epub 2008/10/07. 10.1016/j.ajic.2007.12.008 . 18834732

[26] DeMaio TJ. Social desirability and survey. Surveying subjective phenomena. 1984;2:257.

[27] Contzen N, De Pasquale S, Mosler H-J. Over-Reporting in Handwashing Self-Reports: Potential Explanatory Factors and Alternative Measurements. PloS one. 2015;10(8):e0136445 10.1371/journal.pone.0136445 26301781 PMC4547747

