# Supplementary material for: Measuring office workplace interactions and hand hygiene behaviors through electronic sensors: A feasibility study
Source: PLoS One. 2021 Jan 19;16(1):e0243358. doi: 10.1371/journal.pone.0243358 (PMC7815125; doi:10.1371/journal.pone.0243358)
Supplement: S1 Data — (DOCX) [file pone.0243358.s004.docx]

**Measuring office workplace interactions and hand hygiene behaviors through electronic sensors: a feasibility study**

Paul N. Zivich, Will Huang, Ali Walsh, Marisa Eisenberg, Allison E. Aiello

*Supplementary File 1: Further details regarding sensors*

Opo

Opo sensors collect contact interactions or contacts with other Opos when the front of sensors are facing each other via ultrasonic (40,000 Hz) frequencies. Opo only interacts with other Opo sensors, meaning that it is an opt-in system. Furthermore, the Opo signals do not travel through walls.

To measure interaction distance, Opo utilizes the time difference in ultrasonic / radio signal arrival. Like estimating the distance to a storm based on the time between a lightning flash and subsequent thunder strike, Opo uses the time difference between simultaneously transmitted radio packet and ultrasonic pulse. Radio signals travel at the speed of light, causing them to arrive at nearby Opo sensors almost instantaneously; whereas ultrasonic pulses travel at the speed of sound. Opo sensors receive both the radio signal and ultrasonic pulse and use the time difference between the arrival of the two signals and the speed of sound to accurately determine their distance from the transmitter. Opo provides 5 centimeters resolution for interaction distances up to 2 meters.

| **Supplemental Figure 1: Implemented Opo sensor** |
| --- |
| 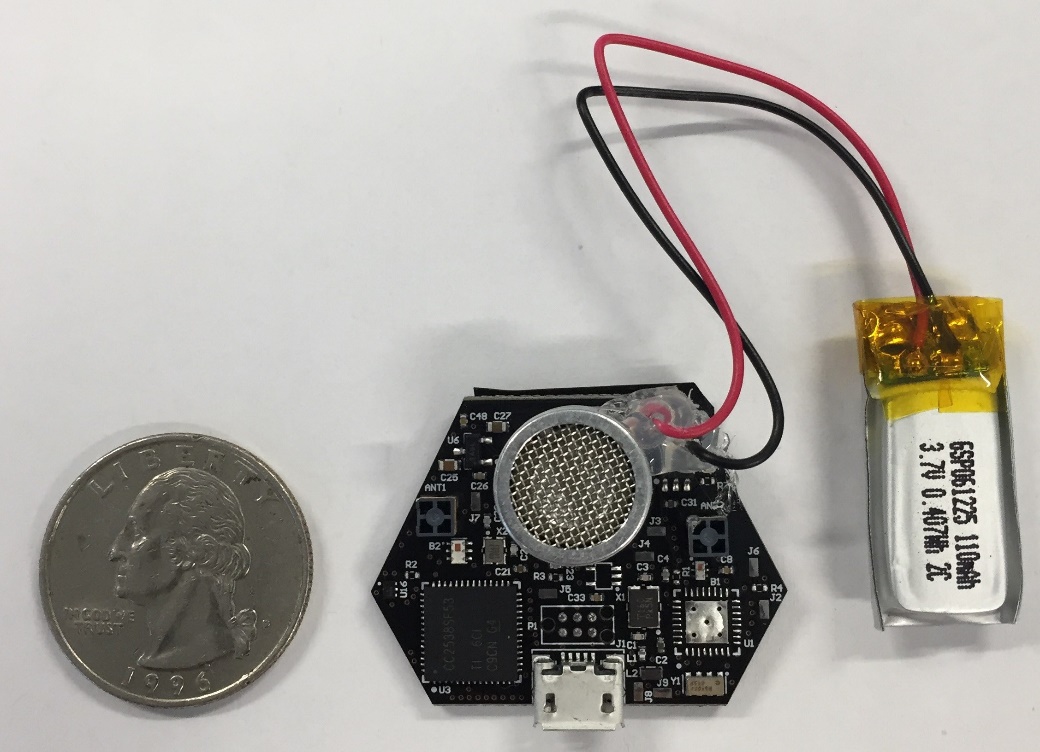 |
| Quarter provided for size reference |

| **Supplemental Figure 2: Visualization of Interactions recorded by Opo sensors and the data downloading process** |
| --- |
| 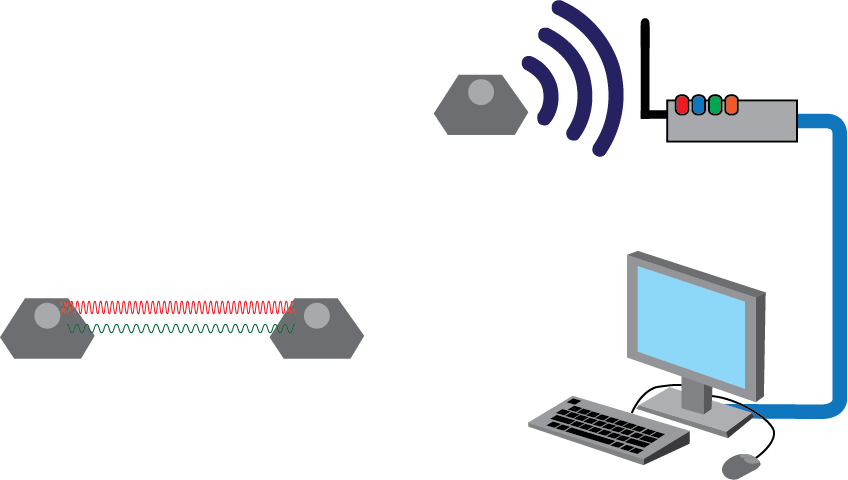 |
| Interactions are captured when two Opos face each other (whether they are affixed to environmental objects or on individuals). Data is downloaded from the sensor to a gateway. Data can then be downloaded from the gateway to a computer. |

BLE Beacons

When a participant in enrolled in the study, they are given a BLE beacon (about the size of a half dollar / 0.5in thick; Supplemental Figure 3) that has a unique identifier consisting of a UUID, Major ID, and Minor ID. This ID is generated based on the Ethica generated study number and the participant’s assigned ID. During the study, the beacon given to participants broadcasts its ID at a set rate (10 Hz).

To capture the broadcast beacon IDs, participants will be asked to download the Ethica data smartphone application. Smartphones running the Ethica application read incoming BLE ID and check whether the UUID is a valid ID for the study (Supplemental Figure 4). If it is a valid ID, the smartphone records the identifiers of the beacon, the received signal strength indicator (RSSI), and the time. Unlike the Opo sensor, the BLE signals travel through walls. Therefore, false negative contacts are more possible (but can be reduced by filtering on recorded RSSI values). Data is sent from the smartphone to the Ethica servers through a wireless connection (Wi-Fi or cell signal). Recorded data is downloaded directly from the Ethica servers by researchers.

| **Supplemental Figure 3: BLE Beacon next to a phone** |
| --- |
| 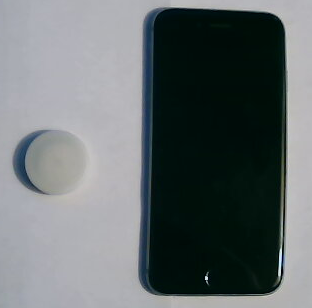 |

| **Supplemental Figure 4:** **Visualization of Interactions recorded by BLE beacons and the Ethica application** |
| --- |
| 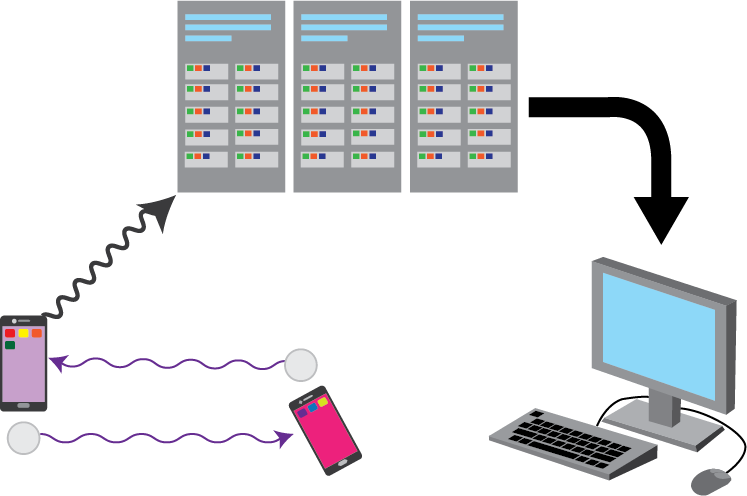 |
| Phones running the Ethica smartphone application passively capture broadcast BLE signals from beacons. Data is stored locally on the phone until uploaded to the Ethica servers. The captured data can then be downloaded from the Ethica servers. |
